# Harnessing Rhizobia to Improve Heavy-Metal Phytoremediation by Legumes

**DOI:** 10.3390/genes9110542

**Published:** 2018-11-08

**Authors:** Camilla Fagorzi, Alice Checcucci, George C. diCenzo, Klaudia Debiec-Andrzejewska, Lukasz Dziewit, Francesco Pini, Alessio Mengoni

**Affiliations:** 1 Department of Biology, University of Florence, Via Madonna del Piano 6, 50019 Sesto Fiorentino, Italy; camilla.fagorzi@unifi.it (C.F.); georgecolin.dicenzo@unifi.it (G.C.D.); 2 Laboratory of Environmental Pollution Analysis, Faculty of Biology, University of Warsaw, Miecznikowa 1, 02-096 Warsaw, Poland; k.debiec@biol.uw.edu.pl; 3 Department of Bacterial Genetics, Institute of Microbiology, Faculty of Biology, University of Warsaw, Miecznikowa 1, 02-096 Warsaw, Poland; ldziewit@biol.uw.edu.pl; 4 Department of Agri-food Production and Environmental Science, University of Florence, 50144 Florence, Italy; francesco.pini@unifi.it

**Keywords:** soil bioremediation, heavy-metals, serpentine soils, serpentine vegetation, genome manipulation, *cis*-hybrid strains

## Abstract

Rhizobia are bacteria that can form symbiotic associations with plants of the Fabaceae family, during which they reduce atmospheric di-nitrogen to ammonia. The symbiosis between rhizobia and leguminous plants is a fundamental contributor to nitrogen cycling in natural and agricultural ecosystems. Rhizobial microsymbionts are a major reason why legumes can colonize marginal lands and nitrogen-deficient soils. Several leguminous species have been found in metal-contaminated areas, and they often harbor metal-tolerant rhizobia. In recent years, there have been numerous efforts and discoveries related to the genetic determinants of metal resistance by rhizobia, and on the effectiveness of such rhizobia to increase the metal tolerance of host plants. Here, we review the main findings on the metal resistance of rhizobia: the physiological role, evolution, and genetic determinants, and the potential to use native and genetically-manipulated rhizobia as inoculants for legumes in phytoremediation practices.

## 1. Introduction 

Plants are colonized by an extraordinarily high number of (micro)organisms, which may reach numbers much larger than those of plant cells [1]. This is particularly evident in the rhizosphere, the thin layer of soil surrounding and influenced by plant roots, where a staggering diversity of microorganisms is present. The collective communities of plant-associated microorganisms are referred to as the plant microbiota, and include the microbial communities of the rhizosphere, as well as those of the external and internal (the endosphere) plant tissues (for examples see [1,2,3,4]). The rhizobiome refers specifically to the microbial community of the rhizosphere, and microbes from this community have been deeply studied for their beneficial effects on plant growth and health. These mainly include mycorrhizal fungi (AMF) and plant-growth promoting rhizobacteria (PGPR), with the latter including the nitrogen fixing legume endosymbiotic bacteria known as rhizobia [5]. Rhizobia are a paraphyletic group of nitrogen fixing bacteria belonging to the Alpha- and Betaproteobacteria classes. Rhizobia can penetrate plant tissues and establish an intracellular population within specialized tissue (known as a nodule) on the root (or stem in a few cases) of leguminous plants. Once inside the cells, the rhizobia differentiate into forms known as bacteroids, which are able to perform nitrogen fixation (the formation of ammonia from di-nitrogen gas) [6]. This process, termed “symbiotic nitrogen fixation” (SNF), provides the plant with nitrogen to sustain its growth in nitrogen-deficient soils, and has been suggested as one of the factors contributing to the evolutionary success of the Fabaceae plant family [6]. Plant growth and crop yield in agricultural systems emerge as the net results of the interactions between the specific plant cultivar and its associated microbiome [7].

**Figure 1:**
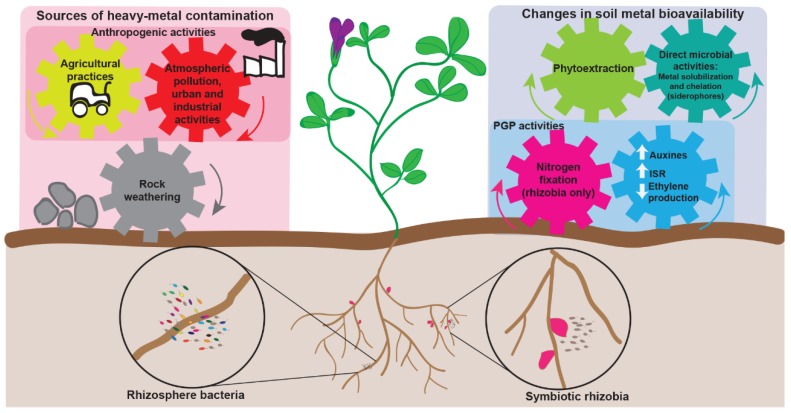
The multiple roles of bacteria in helping plants cope with heavy metals. Plant-associated bacteria may have various roles in both phytostabilization and plant growth. They may influence metal solubility by directly producing molecules for metal chelation (e.g., siderophores), or by influencing plant root growth, resulting in increased production of root exudates. Moreover, both rhizospheric and endophytic bacteria can positively affect plant growth by producing phytohormone molecules (e.g., auxins), alleviating plant stress (e.g., plant ethylene production), or through nitrogen fixation. This latter activity is especially relevant when leguminous plants and their rhizobial microsymbionts are considered. PGP: Plant growth promotion.

Heavy metals are naturally present in soils; however, their increase over certain thresholds has become a worldwide issue [8]. The major cause of heavy-metal contamination in soil is anthropogenic activities (i.e., atmospheric pollution, industrial and urban waste, mining, and some agricultural practices), while natural contamination is mainly due to weathering of metal-enriched rocks [9]. Plant-associated microbiomes play important roles in phytoremediation, allowing plants to thrive on contaminated soils, alleviating the stress associated with toxic levels of heavy-metals and metalloids (such as As), and increasing phytoextraction and phytostabilization [10,11,12,13,14]. Phytoextraction refers to the plants’ ability to import soil contaminants through their roots, and to accumulate these compounds in the aboveground tissues [15]. Phytostabilization involves the immobilization of pollutants in the soil as a result of either their absorption and accumulation in the roots, their adsorption on the root surface, or their transformation within the rhizosphere into sparingly-soluble compounds [16]. In plants such as legumes, which are generally non-hyperaccumulating species, phytostabilization is likely the more relevant process when considering the remediation of contaminated soils [15,16,17]. Plant-associated bacteria may promote the chemical transformation, the chelation, or precipitation and sorption of heavy-metals [18] (Figure 1). For instance, some endophytic bacteria may reduce heavy-metal toxicity [19,20]. Improved growth and increased chlorophyll content were detected in several crop plants inoculated with siderophore-producing bacteria [19]. Additionally, enhanced plant biomass production and remediation has been observed in several hyperaccumulating plants following inoculation with rhizosphere or endophytic bacteria with plant growth promoting (PGP) capabilities [21], such as 1-aminocyclopropane-1-carboxylate (ACC) deaminase production (for detailed reviews, please see [11,12]).

The association between leguminous plants and symbiotic rhizobia has stirred the attention of researchers involved in the restoration of heavy-metal-contaminated sites [22]. The possibility to cultivate legumes on marginal and nutrient-poor soils thanks to the intimate association with PGPR, particularly with nitrogen-fixing rhizobia, has been seen as an opportunity to increase phytoremediation efficiencies while simultaneously reducing its costs [23]. Heavy-metals play central roles in symbiotic nitrogen fixation (see [24] for a review of on the role of metals in the symbiosis). Notably, the nitrogenase enzyme is dependent on a cofactor containing molybdenum and iron (FeMo-co), vanadium and iron (VFe-co), or two iron molecules (FeFe-co). There is also evidence for the role of nickel in the symbiosis. For instance, plants inoculated with a deletion mutant of the rhizobium *Sinorhizobium meliloti* lacking the *nreB*-encoded Ni^2+^ efflux system displayed increased growth under controlled conditions [25]. Additionally, a treatment with low doses of Ni^2+^ as the amendment was shown to stimulate nitrogen fixation and plant growth in soybean, and to increase hydrogenase activity in *Rhizobium leguminosarum* bv. *viciae* [26,27]. However, an excess of heavy-metals negatively impacts the symbiosis, reducing the number of symbiotic nodules, the rate of nodulation, and the rate of nitrogen fixation [28,29]. Consequently, in order to promote legume-based phytoremediation through the improvement of the host plant-symbiont partnership, there is a need to discover metal-resistant rhizobia and/or to manipulate existing rhizobial inoculants to increase their level of metal resistance.

In this review, we summarize the main findings on metal resistance in rhizobia: the physiological role, evolution, and genetic determinants of metal resistance, and the perspective to use native and genetically-manipulated rhizobia as inoculants for legumes in phytoremediation practices.

## 2. Legumes in Heavy-Metal Contaminated Areas

The family Leguminosae (Fabaceae) is one of the most diverse among land plants and includes over 700 genera and 20,000 species [30]. Legumes have been proposed as relevant species for phytoremediation, largely due to their ability to colonize marginal lands and nutrient-poor soils [28,31]. In particular, legumes are relevant for phytostabilization, as only a few species have been found to be metal hyperaccumulators (e.g., some species of the genus *Astragalus* isolated in the Western United States are selenium hyperaccumulators) for phytoextraction [23,28,32]. Normally, the symbiosis with rhizobia is inhibited by high levels of heavy-metals in the soil, and genetic engineering techniques have been suggested to improve symbiotic nitrogen fixation under such harsh environmental conditions [33]. However, although such biotechnological proposals are interesting in terms of molecular dissection of the system and theoretical application, currently, there are a number of limitations to the use of genetically-modified microorganisms, including their free release in nature. Analyses on legumes from heavy-metal-contaminated soils have led to the discovery of naturally-resistant rhizobia, which could be used as inoculants in these extreme environments. However, a deeper investigation of leguminous plants growing in metal-enriched sites is required to improve legume-based phytoremediation.

### 2.1. The Serpentine Vegetation: A Source of Legumes Evolved on Heavy-Metal Rich Soils

Serpentine rocks are an array of ultramafic rock types composed of a hydrous magnesium iron phyllosilicate mineral that originates from metamorphic alterations of peridotite and pyroxene with water. The soils derived from these rocks are characterized by: (i) high levels of nickel, cobalt, and chromium, (ii) low levels of N, P, K, and Ca, and (iii) a high Mg/Ca ratio [34]. This chemical composition strongly limits the growth of most plant species [35], as well as many microorganisms [3]. The presence of serpentine outcrops is scattered across the planet. Along a geological timescale, serpentine outcrops have prompted the evolution of peculiar plant adaptation mechanisms (such as metal hyperaccumulation [36]), which then gave rise to plant differentiation and speciation in a classical “ecological islands” model [37,38]. Serpentine vegetation in temperate ecosystems includes several leguminous species from various genera, including *Lotus*, *Lupinus*, *Trifolium*, *Vicia*, *Melilotus*, *Medicago*, *Lathyrus*, *Ononis*, *Dorychnium*, *Chamaecytisus*, *Astragalus*, *Anthyllis*, *Cytisus*, and *Acmispon* [39,40]. Serpentine endemic legumes have also been reported, such as *Errazurizia benthamii* [41] in North America, and *Serianthes calycina* [42] in New Caledonia. The microbiomes associated with serpentine plants contain a fraction of microorganisms that appear to have specifically evolved functions to cope with toxic levels of metals present in the soil and in the plant itself [3]. Moreover, some of these microorganisms have been shown to be effective in promoting host plant growth in serpentine soil and, for metal hyperaccumulating plants, to increase metal translocation to the aerial part [43]. Consequently, rhizobia from serpentine endemic legumes (such as Ni-resistant bradyrhizobia from *S. calycina* [42]) may already be adapted to optimizing the fitness of their host in serpentine environments through a long-term natural selection process [44]. Serpentine endemic legumes may therefore represent an ideal source of rhizobia that are naturally highly-competent symbiotic partners in heavy-metal contaminated soils. 

### 2.2. The Search for Heavy-Metal Tolerant Rhizobia and Their Use as Inoculants

Legumes growing in contaminated areas such as mine deposits and serpentine soils have been a source of symbiotic rhizobial strains displaying resistance to heavy-metals, including Zn, Pb, and Cu [45,46,47,48]. Table 1 summarizes the main studies on the (positive) effects of rhizobial inoculation on the heavy-metal tolerance of host plants. 

*Anthyllis vulneraria* is one of the most relevant legumes for isolating rhizobia that promote metal-tolerance by the host plant. *A. vulneraria* is a perennial herb from boreo-temperate climate areas in Europe, and it can be found colonizing rocky outcrops and establishing populations on heavy-metal (mainly Zn)-contaminated sites. *Anthyllis* is characterized by determinate nodules, where the meristematic activity disappears shortly after nodule formation, resulting in nodules of spherical shape. *Anthyllis* nodules contain a multilayer cortex: a glycoproteic parenchyma for diffusion, an endodermis, and the outer cortex, which mainly serves as a barrier against pathogens [49]. Nodule bacterial population of leguminous plants grown in Morocco metal-polluted soil displayed a great biodiversity, suggesting that, in these conditions, metal resistant non-rhizobia may efficiently colonize the nodules as endophytes [50]. This highlights the importance of heavy-metal resistance in rhizobia for the establishment of an effective symbiotic interaction in contaminated soils. *A. vulneraria* has been found to be associated with rhizobial symbionts from the genera *Mesorhizobium*, *Rhizobium*, and *Aminobacter*. These include novel rhizobial species, such as *Mesorhizobium metallidurans*, *Rhizobium metallidurans*, and *Aminobacter anthyllidis* [45,47,48,49,50,51]. Interestingly, these novel rhizobial species have so far been identified only in Pb-contaminated environments and not in unpolluted soils [47]. The symbiosis between *A. vulneraria* and its possibly exclusive metal-resistant bacterial species may provide the basis for the establishment of phytoremediation practices. This could involve the use of *A. vulneraria* metal-resistant germplasms, together with its specific natural rhizobial symbionts. Alternatively, the heavy-metal-resistant rhizobia isolated from *A. vulneraria* could be modified, either through laboratory-based experimental evolution studies [52] or direct genetic manipulation, to be capable of entering into an effective symbiosis with other host legumes.

Legumes of the genus *Medicago* have also been deeply investigated for their application in phytoremediation (see Table 1 and references therein). This is mainly because species from this genus are important forage crops for which cultivation techniques and genetics are well established, providing important advantages for future cost-effective applications [53]. Genetically-modified [54,55] and natural [56,57] inocula of *Sinorhizobium* (syn. *Ensifer*) *meliloti* and *Sinorhizobium medicae* [54] have been examined for their abilities to improve plant growth and metal accumulation in the presence of toxic levels of heavy metals such as Cu, Cd, and Zn. However, genetic manipulation is not absolutely required, as interesting results have also been obtained using indigenous *S. meliloti* and *S. medicae* strains directly isolated from contaminated soils [56,57]. For example, inoculation of *Medicago sativa* plants, grown under field conditions, with wild *S. meliloti* and *S. medicae* strains resulted in active nodulation and the promotion of metal bioaccumulation within the root nodules [56,57]. These results suggest that the exploitation of natural rhizobia could be a valuable tool for promoting land restoration and phytostabilization by legumes.

Legume-based phytoremediation may also be improved through inoculation with a consortium of metal-resistant rhizobia and other PGP bacteria. In metal polluted soil, inoculation of *Lupinus luteus* with *Bradyrhizobium* sp. 750 in consortium with *Pseudomonas* sp. Az13 and *Ochrobactrum cytisi* Azn6.2 increased plant biomass by greater than 100% with respect to uninoculated plants [10]. In contrast, inoculation with only *Bradyrhizobium* sp. 750 increased plant biomass by only 30%. Similarly, co-inoculation of *M. lupina* with *S. meliloti* CCNWSX0020 and *Pseudomonas putida* UW4 resulted in larger plants and greater total Cu accumulation than inoculation with just *S. meliloti* CCNWSX0020 [55]. Inoculation of *Vicia faba*, *Lens culinaris*, and *Sulla coronaria* with consortia of rhizobia and non-rhizobia was also effective at improving plant growth and pod yield when grown in metal-contaminated soil [58]. Moreover, the inoculated *S. coronaria* accumulated significantly more cadmium than uninoculated plants [58]. These results highlight the potential for root-associated microbial communities to influence the success of phytoremediation by rhizobium-inoculated legumes.

It may be concluded that there is great biotechnological potential in increasing the phytoremediation capabilities of legumes by their associated rhizobia. This may be mediated through at least two mechanisms: (i) reducing the toxic effects of the metals, and (ii) promoting the growth of the plant through PGP activities.

## 3. Genetics and Genomics of Heavy-Metal Resistance in Symbiotic Rhizobia 

A deep understanding of the genetics and molecular mechanisms of metal resistance remains one of the main goals in environmental biotechnology, with the final aim of promoting the bioremediation (including phytoremediation) of contaminated soils. Table 2 reports the main studies evaluating the genetic determinants of heavy metal resistance in rhizobia. Such studies have most commonly identified the presence of efflux systems that increase metal tolerance by reducing the intracellular concentrations of the metal(s). However, studies employing genome-scale methods, such as transcriptome analyses and transposon mutagenesis, have demonstrated that the cellular response to metal stress involves an intricate genetic network.

Mechanisms mediating resistance to Co and Ni have been identified in many metal resistant rhizobia through the identification of orthologs of metal resistance genes characterized in *Cupriavidus metallidurans* CH34 [63,64]. A gene encoding a DmeF ortholog has been identified in *R. leguminosarum* bv. *viciae* strain UPM791 [65]. DmeF proteins belong to the cation diffusion facilitator (CDF) protein family, which form metal/proton antiport systems to translocate heavy metals across the bacterial membrane [66]. Mutation of the *dmeRF* operon in *R. leguminosarum* resulted in increased sensitivity to Co and Ni, but not to Zn or Cu [65]. The mutant also appeared to be somewhat less effective in symbiosis with pea plants, but not lentil plants, when grown with high concentrations of Co or Ni [65]. Further experiments demonstrated that *dmeR* encodes a Ni- and Co-responsive transcriptional regulator that represses expression of the efflux system in the absence of these metals [65]. Despite being considered a metal-sensitive strain, the *S. meliloti* strain 1021 encodes various metal homeostasis mechanisms, including the DmeRF system, several P-ATPases that are highly common in bacteria, and an ortholog of the *C. metallidurans* NreB protein [25,65,67]. Mutation of *nreB*, encoding a Ni^2+^ efflux protein, resulted in increased sensitivity to Ni, Cu, and low pH, but increased tolerance to urea osmotic stress [25]. The P_1B-5_-ATPase of *S. meliloti*, termed Nia (nickel iron ATPase), is positively induced by the presence of Ni^2+^ and Fe^2+^, and its expression is higher within nodules relative to free-living cells, which may prevent toxic levels of iron accumulation in the symbiosomes. The wild type protein and recombinants with a deletion of the C-terminal Hr domain have been used to understand the metal specificity of the P_1B-5_-ATPase family [67].

Genome-wide analyses have been used to investigate the genetics of the resistance mechanisms in *S. meliloti* strain CCNWSX0020, which is resistant to high levels of various heavy-metals (Cu, Zn, Cd and Pb). Gene mutation and transcriptome analyses have suggested the involvement of dozens of genes in the metal-resistance phenotypes of CCNWSX0020, including housekeeping genes [68,69,70]. Of particular note are the following three operons: the multicopper oxidase (MCO), CopG, and YadYZ operons. The MCO operon is highly expressed following exposure to Cu, and it encodes an outer membrane protein (Omp), the multicopper oxidase CueO, a blue copper azurin-like protein, and a copper chaperone involved in Cu homeostasis [70]. It was proposed that the CueO protein (showing 40% similarity with the CueO protein of *E. coli*) catalyzes Cu(I) oxidation in the periplasmic space, followed by the export of the excessive Cu(II) across the outer membrane [70,71]. The CopG operon consists of four genes: CopG, a CusA-like protein, a FixH-like protein, and a hypothetical protein. Mutation of any of the latter three genes resulted in elevated sensitivity to Zn, Pb, Cd, and Cu, although the mechanism of resistance of this operon remains unknown [70]. The CusA-like protein appears to be a highly-truncated ortholog of the CusA protein of the CusCBA Cu(I) efflux system of *E. coli* [72,73], and may act as a metal binding protein [70]. The FixH-like protein displays similarity to the FixH protein of the FixHGI membrane-bound system, a likely cation transporter that has been shown to be essential for symbiotic nitrogen fixation [74,75]. A FixH-like homolog is also encoded by the pSinB plasmid of *Ensifer* sp. M14 (formerly *Sinorhizobium* sp. M14), where it was also experimentally shown to be involved in metal resistance [76]. Deletion of the *yedYZ* operon resulted in increased sensitivity to Zn, Pb, Cd, and Cu [70]. This was the first report suggesting that YedYZ may be involved in heavy-metal tolerance. In *E. coli*, YedYZ forms a sulfite oxidoreductase [77], and expression of a homologous protein in *S. meliloti* 1021 is induced by taurine and thiosulfate [78]. Thus, the heavy-metal resistance phenotype may be mediated through disrupting sulfite metabolism, which may influence antioxidant defenses against reactive oxygen species (ROS) generated by heavy metals [70].

Many scientists have used population genetics approaches to identify loci associated with heavy-metal resistance. This was achieved by performing genome-wide association studies on a population’s pan-genome, considering allelic variations in the core genome (the set of genes shared by the members of the population), and gene presence/absence in the dispensable genome fraction (the set of genes present in only a fraction of the population). Genomic variants statistically associated with nickel adaptation were identified in a *Mesorhizobium* population using this approach [79]. A population of 47 *Mesorhizobium* strains, isolated from root nodules and soils with different levels of nickel contamination, was studied. Most of the variants associated with metal adaptation were found in the dispensable genome fraction. This work highlights that adaptation to heavy metal stress is likely driven predominately by horizontal gene transfer, and is not due to mutations of pre-existing genes.

Multiple studies have demonstrated that the genetic determinants of metal-resistance in rhizobia are relevant for phytoremediation purposes. Mutation of *ceuO*, *yedYZ*, and the *fixH*-like gene negatively impacted the *M. lupulina* nodulation kinetics of *S. meliloti* CCNWSX0020 in the presence of Cu and/or Zn [70], while deletion of the *cusA*-like gene had a negative effect, even in the absence of heavy metals. It was separately observed that *M. lupulina* plants inoculated with *S. meliloti* CCNWSX0020 strains with independent mutations in five Cu resistance loci were smaller than plants inoculated with the wild type, when grown in the presence of Cu [80]. Notably, *M. lupulina* plants inoculated with any of the *S. meliloti* CCNWSX0020 mutants mentioned above accumulated lower amounts of Cu and/or Ni [78]. Similarly, *Robinia pseudoacacia* plants inoculated with a *Mesorhizobium amorphae* 186 *copA* mutant accumulated 10–15% less Cu than plants inoculated with the wild type [81]; however, no effect on plant growth was observed.

## 4. Genomic Manipulation Strategies for Improving Legume Phytoremediation

Various attempts have been made to increase plant growth in the presence of toxic metal concentrations through genetic modification of their rhizobial microsymbionts. One approach is to introduce new genes conferring heavy-metal resistance into the rhizobium. For example, inoculation of a genetically-modified *M. truncatula* line (which expressed a metallothionein gene from *Arabidopsis thaliana* in its roots) with wild type *S. medicae* resulted in elevated Cu tolerance [84]. Copper tolerance was further increased using a *S. medicae* strain expressing the *P. fluorescence copAB* Cu resistance genes [84]. Inoculation with the latter strain also resulted in elevated Cu accumulation in the plant roots [84]. Similarly, the introduction of an algal As(III) methyltransferase gene (*arsM*) into the chromosome of *R. leguminosarum* bv. *trifolii* produced a strain that was able to methylate and volatilize inorganic arsenic in symbiosis with red clover (with no negative impact on nitrogen fixation) [85]. A second approach is the insertion of genes in rhizobia to modulate phytohormone production, thereby reducing plant stress perception. For example, an ACC deaminase overproducing *S. meliloti* strain increased Cu tolerance and promoted plant growth of the host plant *M. lupulina* [86]. This result was probably due to reduced production of ethylene by the host plant, in turn decreasing stress perception. However, it should be kept in mind that a relatively high number of genes may contribute to the heavy-metal stress response [87,88,89]. Consequently, a multigenic, genome-wide approach should be considered when attempting to genetically modify competitive rhizobial symbionts to have increased heavy-metal tolerance. One possibility along these lines is the introduction of entire, large resistance plasmids from a non-symbiotic (but highly resistant) strain to a phylogenetically-related, symbiotic metal-sensitive strain. A candidate plasmid for such studies is the pSinA plasmid of the non-symbiotic *Ensifer* sp. M14, which was isolated from an As-contaminated gold mine [76,90,91]. The pSinA plasmid is a self-transmissible replicon with a broad host range. It harbors a genomic island with genes for arsenite oxidation (*aio* genes) and arsenite resistance (*ars* genes), and its transfer to other species results in increased arsenic resistance [90]. Transfer of the pSinA plasmid to closely-related rhizobia, such as *S. meliloti*, may result in the construction of As-tolerant legume symbionts for use in arsenic remediation. Subsequent acquisition of pSinA by other members of the rhizospheric microbiota may further stimulate phytoremediation of arsenic contaminated soils through reducing the arsenic toxicity (oxidizing arsenites to arsenates) and biofortification (increase of the arsenic resistance level) of the autochthonic or augmented microflora.

**Figure 2:**
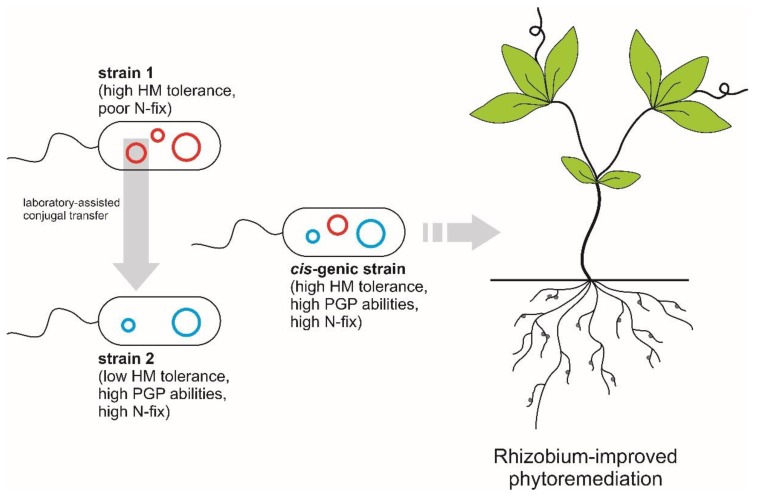
A synthetic biology-based proposal to increase rhizobial-mediated heavy-metal tolerance. Surveys of rhizobial phenotypic and genetic diversity in heavy-metal (HM) rich areas facilitates the discovery of strains (strain 1) with high levels of heavy-metal resistance. However, such strains may not be competitive or good nitrogen-fixers in the crops to be used for phytoremediation. The simultaneous transfer of a large collection of genomic determinants that contribute to HM tolerance, good PGP, and/or nitrogen fixation (N-fix) abilities between two or more strains (strain 2) could create hybrid strains (*cis-*genic strain) with improved features for application in the field for phytoremediation.

Similarly, elite and metal-resistant rhizobia may be obtained through combining within one strain genomic elements from the species pangenome. The genomes of most rhizobia are extremely diverse, and many rhizobia have a divided genome structure consisting of at least two large DNA replicons [92]. Although there can be numerous inter-replicon functional, regulatory, and genetic interactions [93,94,95], in some ways, each replicon in a divided genome could be considered as an independent evolutionary and functional element [94,96,97,98]. Recently, it was shown that the genome and metabolism of *S. meliloti* is robust to the replacement of the symbiotic megaplasmid with the symbiotic megaplasmid of a different wild-type isolate [99]. Therefore, it may be possible to construct “hybrid” strains (Figure 2) with a collection of replicons derived from various wild-type isolates, potentially allowing for the development of elite strains with improved multifactorial phenotypes (e.g., resistance to heavy-metals, high symbiotic efficiency, and competition toward the indigenous soil microbiota). 

## 5. Conclusions

In recent years, the number of studies related to the potential exploitation of rhizobium–legume symbioses for phytoremediation practices have increased enormously as a result of environmental emergencies. In this brief review, we have presented state-of-the-art studies on heavy-metal tolerant rhizobia, and on their applications in phytoremediation as legume symbionts. A large number of investigations have indicated that rhizobia, and especially heavy-metal resistant rhizobia, can increase legume heavy-metal tolerance and promote improved legume growth in metal-rich soils, thereby resulting in greater removal of heavy-metals from the soil. Heavy-metal resistant rhizobia have been isolated from the nodules of legumes grown in soils that are rich in heavy-metals as a result of geological (e.g., serpentine outcrops) or anthropic causes (e.g., mine deposits). Genetic and genomic studies of heavy-metal resistant rhizobia have shown that although relatively few genes act as the main player in tolerance, a much larger set of genes may be involved in maximizing fitness in heavy metal rich growth conditions. Some of these genes, such as the systems for Ni^2+^ efflux in *S. meliloti*, may also contribute to a linkage between metal homeostasis and nitrogen-fixation efficiency. As such, systems-biology approaches are required to develop an overall picture of heavy-metal resistance and the ways that we can increase and exploit it in biotechnology. It will also be important to keep in mind that the engineering of rhizobia should consider several additional aspects, including the rhizobial genotype, the host plant genotype, and the interactions between the rhizobium with the soil and root microbiota [100].

Going forward, we suggest that large-scale genome-manipulation approaches may be considered in developing rhizobial strains with elite phenotypes (e.g., high heavy-metal resistance, high nitrogen-fixation ability, high competitiveness, etc.) for use in phytoremediation applications. As a pre-requisite to such studies, it will be necessary to increase efforts at creating culture collections of rhizobial strains from contaminated areas, since the strains isolated from these environments is quite limited in number and in terms of host plant (see also [23]). Such efforts would benefit from exploring areas that have evolved peculiar flora, such as serpentine outcrops, maximizing the chance to find well-adapted strains. Whole genome sequencing, genome-scale mutagenesis (such as Tn-seq or INseq [101]), and metabolic modeling of these strains could then be employed to fully characterize the genomic basis for tolerance against the contaminants.

## Figures and Tables

**Table 1:** Studies of phytoremediation mediated by rhizobium-inoculated legumes. NA, not analyzed.

Legume Species	Heavy-Metals in the Soil	Rhizobium Inoculant	Co-Inoculation with Other PGPR?	Evidence for Stimulation of Rhizosphere Microbiota	Type of Study	Effect	Reference
*Glycine max*	As	*Bradyrhizobium* sp. Per 3.61	No	NA	Lab scale (pot)	Reduce translocation factor	[59]
*Lupinus luteus*	Cu, Cd, Pb	*Bradyrhizobium* sp. 750	Yes	NA	*In situ*	Increased metal accumulation in root	[10]
*Medicago lupulina*	Cu	*Sinorhizobium meliloti* CCNWSX0020	No	NA	*In vitro* (pot)	Increased plant growth and copper tolerance	[55]
*Medicago sativa*	Cu	*Sinorhizobium meliloti* CCNWSX0020	No	NA	*In vitro*	Increased tolerance of seedlings	[60]
*Medicago sativa*	Cd	*Sinorhizobium meliloti* (from contaminated soil [61])	No	NA	Lab scale (pot)	Increased Cd phytoextraction	[56]
*Medicago sativa*	Zn	*Sinorhizobium meliloti* (from contaminated soil [61])	No	NA	Lab scale (pot with sterile sand)	Increased Zn accumulation in root	[57]
*Medicago truncatula*	Cu	*Sinorhizobium medicae* MA11 (genetically modified with *copAB* genes)	No	NA	*In vitro*	Increased metal accumulation in root	[54]
*Robinia pseudoacacia*	Cd, Zn, Pb	*Mesorhizobium loti* HZ76	No	Yes	Lab scale (pot)	Increased growth of the plant	[62]
*Sulla conoraria*	Cu, Zn, Pb	*Rhizobium sullae*	Yes	NA	*In situ*	Increased soil Zn stabilization	[58]
*Vicia faba*	Cu, Zn, Pb	*Rhizobium* sp. CCNWSX0481	Yes	NA	*In situ*	Increased soil Cu stabilization	[58]

**Table 2:** Genes for heavy-metal (and metalloid) tolerance in symbiotic rhizobia. A summary of the main genes whose function in tolerance was confirmed experimentally is reported.

Strain	Host Plant	Isolation Site	Method of Identification	Gene(s)	Metal(s) Tolerance	Reference
*Bradhyrhizobium* spp.	*Serianthes calycina*	Serpentine (New Caledonia)	PCR amplification, site-directed mutagenesis	*cnr*/*nre* systems	Co, Ni	[42]
*Mesorhizobium* spp.	*Acmispon wrangelianus*	Serpentine (California)	Association mapping	Various	Ni	[79]
*Mesorhizobium metallidurans*	*Antyllis vulneraria*	Zinc mine (France)	Cosmid library	*cadA* (PIB-2-type ATPase)	Zn, Cd	[82]
*Sinorhizobium meliloti* 1021	*Medicago sativa*	Laboratory strain	Site-directed gene deletion	*nreB* (SMa1641)	Ni	[25]
*Sinorhizobium meliloti* 1021	*Medicago sativa*	Laboratory strain	Tn5 insertion, biochemical characterization	SMa1163 (P1B-5-ATPase)	Ni, Fe	[67]
*Sinorhizobium meliloti* CCNWSX0020	*Medicago lupulina*	Mine tailings (China)	Site-directed gene deletion and transcriptomics	P1B-type ATPases and others	Cu, Zn	[69,70]
*Rhizobium leguminosarum* bv. *viciae* UPM1137	*Pisum sativum*	Serpentine (Italy)	Transposon mutagenesis	14 loci (gene annotation corresponds to Rlv 3841 genome): RL2862, RL2436, RL2322, pRL110066, RL1351, RL4539, pRL90287, RL4188, RL2793, RL2100, RL0615, RL1589, pRL110071, RL1553	Ni, Co	[83]

## References

[1] Mendes R., Garbeva P., Raaijmakers J.M. (2013). The rhizosphere microbiome: Significance of plant beneficial, plant pathogenic, and human pathogenic microorganisms. FEMS Microbiol. Rev..

[2] Bai Y., Müller D.B., Srinivas G., Garrido-Oter R., Potthoff E., Rott M., Dombrowski N., Münch P.C., Spaepen S., Remus-Emsermann M. (2015). Functional overlap of the *Arabidopsis* leaf and root microbiota. Nature.

[3] Mengoni A., Schat H., Vangronsveld J. (2010). Plants as extreme environments? Ni-resistant bacteria and Ni-hyperaccumulators of serpentine flora. Plant Soil.

[4] Pini F., Frascella A., Santopolo L., Bazzicalupo M., Biondi E.G., Scotti C., Mengoni A. (2012). Exploring the plant-associated bacterial communities in *Medicago sativa* L.. BMC Microbiol..

[5] Nadeem S.M., Ahmad M., Zahir Z.A., Javaid A., Ashraf M. (2014). The role of mycorrhizae and plant growth promoting rhizobacteria (PGPR) in improving crop productivity under stressful environments. Biotechnol. Adv..

[6] Sprent J.I. (2009). Legume Nodulation: A Global Perspective.

[7] Theis K.R., Dheilly N.M., Klassen J.L., Brucker R.M., Baines J.F., Bosch T.C.G., Cryan J.F., Gilbert S.F., Goodnight C.J., Lloyd E.A. (2016). Getting the Hologenome Concept Right: An Eco-Evolutionary Framework for Hosts and Their Microbiomes. Msystems.

[8] Chibuike G.U., Obiora S.C. (2014). Heavy metal polluted soils: Effect on plants and bioremediation methods. Appl. Environ. Soil Sci..

[9] Lebrazi S., Fikri-Benbrahim K. (2018). Rhizobium-Legume Symbioses: Heavy metal effects and principal approaches for bioremediation of contaminated soil. Legumes for Soil Health and Sustainable Management.

[10] Dary M., Chamber-Pérez M.A., Palomares A.J., Pajuelo E. (2010). “In situ” phytostabilisation of heavy metal polluted soils using *Lupinus luteus* inoculated with metal resistant plant-growth promoting rhizobacteria. J. Hazard. Mater..

[11] Kong Z., Glick B.R. (2017). The Role of Plant Growth-Promoting Bacteria in Metal Phytoremediation. Advanced in Microbial Physiology.

[12] Sessitsch A., Kuffner M., Kidd P., Vangronsveld J., Wenzel W.W., Fallmann K., Puschenreiter M. (2013). The role of plant-associated bacteria in the mobilization and phytoextraction of trace elements in contaminated soils. Soil Biol. Biochem..

[13] Weyens N., Lelie D. Van Der, Taghavi S., Newman L. (2009). Exploiting plant—Microbe partnerships to improve biomass production and remediation. Trends Biotechnol..

[14] Kidd P.S., Alvarez-Lopez V., Becerra-Castro C., Cabello-Conejo M., Prieto-Fernandez A. (2017). Potential role of plant-associated bacteria in plant metal uptake and implications in phytotechnologies. Advances in Botanical Research.

[15] Mahar A., Wang P., Ali A., Awasthi M.K., Lahori A.H., Wang Q., Li R., Zhang Z. (2016). Challenges and opportunities in the phytoremediation of heavy metals contaminated soils: A review. Ecotoxicol. Environ. Saf..

[16] Bolan N.S., Park J.H., Robinson B., Naidu R., Huh K.Y. (2011). Phytostabilization: A green approach to contaminant containment. Adv. Agron..

[17] Mahieu S., Frérot H., Vidal C., Galiana A., Heulin K., Maure L., Brunel B., Lefèbvre C., Escarré J., Cleyet-Marel J.-C. (2011). *Anthyllis vulneraria/Mesorhizobium metallidurans*, an efficient symbiotic nitrogen fixing association able to grow in mine tailings highly contaminated by Zn, Pb and Cd. Plant Soil.

[18] Gadd G.M. (2008). Accumulation and transformation of metals by microorganisms. Biotechnology: Special Processes.

[19] Mastretta C., Taghavi S., Van Der Lelie D., Mengoni A., Galardi F., Gonnelli C., Barac T., Boulet J., Weyens N., Vangronsveld J. (2009). Endophytic bacteria from seeds of *Nicotiana tabacum* can reduce cadmium phytotoxicity. Int. J. Phytoremediat..

[20] Etesami H. (2018). Bacterial mediated alleviation of heavy metal stress and decreased accumulation of metals in plant tissues: Mechanisms and future prospects. Ecotoxicol. Environ. Saf..

[21] Novo L.A.B., Castro P.M.L., Alvarenga P., da Silva E.F. (2018). Plant Growth–Promoting Rhizobacteria-Assisted phytoremediation of mine soils. Bio-Geotechnologies for Mine Site Rehabilitation.

[22] Teng Y., Wang X., Li L., Li Z., Luo Y. (2015). Rhizobia and their bio-partners as novel drivers for functional remediation in contaminated soils. Front. Plant Sci..

[23] Checcucci A., Bazzicalupo M., Mengoni A. (2017). Exploiting nitrogen-fixing rhizobial symbionts genetic resources for improving phytoremediation of contaminated soils. Enhancing Cleanup of Environmental Pollutants.

[24] González-Guerrero M., Matthiadis A., Saez Á., Long T. (2014). Fixating on metals: New insights into the role of metals in nodulation and symbiotic nitrogen fixation. Front. Plant Sci..

[25] Pini F., Spini G., Galardini M., Bazzicalupo M., Benedetti A., Chiancianesi M., Florio A., Lagomarsino A., Migliore M., Mocali S. (2013). Molecular phylogeny of the nickel-resistance gene *nreB* and functional role in the nickel sensitive symbiotic nitrogen fixing bacterium *Sinorhizobium meliloti*. Plant Soil.

[26] Lavres J., Castro Franco G., de Sousa Câmara G.M. (2016). Soybean seed treatment with nickel improves biological nitrogen fixation and urease activity. Front. Environ. Sci..

[27] Ureta A.-C., Imperial J., Ruiz-Argüeso T., Palacios J.M. (2005). *Rhizobium leguminosarum* biovar viciae symbiotic hydrogenase activity and processing are limited by the level of nickel in agricultural soils. Appl. Environ. Microbiol..

[28] Hao X., Taghavi S., Xie P., Orbach M.J., Alwathnani H.A., Rensing C., Wei G. (2014). Phytoremediation of heavy and transition metals aided by legume-rhizobia symbiosis. Int. J. Phytoremediat..

[29] Ahmad E., Zaidi A., Khan M.S., Oves M. (2012). Heavy metal toxicity to symbiotic nitrogen-fixing microorganism and host legumes. Toxicity of Heavy Metals to Legumes and Bioremediation.

[30] Doyle J.J., Luckow M.A. (2003). The rest of the iceberg. Legume diversity and evolution in a phylogenetic context. Plant Physiol..

[31] Bradshaw A.D., Chadwick M.J. (1980). The Restoration of Land: The Ecology and Reclamation of Derelict and Degraded Land.

[32] Reeves R.D., van der Ent A., Baker A.J.M. (2018). Global distribution and ecology of hyperaccumulator plants. Agromining: Farming for Metals.

[33] Pajuelo E., Rodríguez-Llorente I.D., Lafuente A., Caviedes M.Á. (2011). Legume–rhizobium symbioses as a tool for bioremediation of heavy metal polluted soils. Biomanagement of Metal-Contaminated Soils.

[34] Brooks R.R. (1987). Serpentine and Its Vegetation: A Multidisciplinary Approach.

[35] Brady K.U., Kruckeberg A.R., Bradshaw H.D. (2005). Evolutionary ecology of plant adaptation to serpentine soils. Annu. Rev. Ecol. Evol. Syst..

[36] Words K. (2010). Metal Hyperaccumulation in plants. Annu. Rev. Plant Biol..

[37] Harrison S., Rajakaruna N. (2011). Serpentine: The Evolution and Ecology of a Model System.

[38] Mengoni A., Mocali S., Surico G., Tegli S., Fani R. (2003). Fluctuation of endophytic bacteria and phytoplasmosis in elm trees. Microbiol. Res..

[39] Alexander E.B., Coleman R.G., Harrison S.P., Keeler-Wolfe T. (2007). Serpentine Geoecology of Western North America: Geology, Soils, and Vegetation.

[40] Pustahija F., Brown S.C., Bogunić F., Bašić N., Muratović E., Ollier S., Hidalgo O., Bourge M., Stevanović V., Siljak-Yakovlev S. (2013). Small genomes dominate in plants growing on serpentine soils in West Balkans, an exhaustive study of 8 habitats covering 308 taxa. Plant Soil.

[41] Selvi F. (2007). Diversity, geographic variation and conservation of the serpentine flora of Tuscany (Italy). Biodivers. Conserv..

[42] Chaintreuil C., Rigault F., Moulin L., Jaffré T., Fardoux J., Giraud E., Dreyfus B., Bailly X. (2007). Nickel resistance determinants in *Bradyrhizobium* strains from nodules of the endemic New Caledonia legume *Serianthes calycina*. Appl. Environ. Microbiol..

[43] Rajkumar M., Narasimha M., Prasad V., Freitas H., Ae N. (2009). Biotechnological applications of serpentine soil bacteria for phytoremediation of trace metals. Crit. Rev. Biotechnol..

[44] Friesen M.L. (2012). Widespread fitness alignment in the legume—Rhizobium symbiosis. New Phytol..

[45] Grison C.M., Jackson S., Merlot S., Dobson A., Grison C. (2015). *Rhizobium metallidurans* sp. nov., a symbiotic heavy metal resistant bacterium isolated from the anthyllis vulneraria Zn-hyperaccumulator. Int. J. Syst. Evol. Microbiol..

[46] Ye M., Liao B., Li J.T., Mengoni A., Hu M., Luo W.C., Shu W.S. (2012). Contrasting patterns of genetic divergence in two sympatric pseudo-metallophytes: *Rumex acetosa* L. and *Commelina communis* L.. BMC Evol. Biol..

[47] Mohamad R., Maynaud G., Le Quéré A., Vidal C., Klonowska A., Yashiro E., Cleyet-Marel J.-C., Brunel B. (2016). Ancient heavy metal contamination in soils as a driver of tolerant *Anthyllis vulneraria* rhizobial communities. Appl. Environ. Microbiol..

[48] Vidal C., Chantreuil C., Berge O., Mauré L., Escarré J., Béna G., Brunel B., Cleyet-Marel J.C. (2009). *Mesorhizobium metallidurans* sp. nov., a metal-resistant symbiont of *Anthyllis vulneraria* growing on metallicolous soil in Languedoc, France. Int. J. Syst. Evol. Microbiol..

[49] Sujkowska-Rybkowska M., Ważny R. (2018). Metal resistant rhizobia and ultrastructure of *Anthyllis vulneraria* nodules from zinc and lead contaminated tailing in Poland. Int. J. Phytoremediat..

[50] El Aafi N., Saidi N., Maltouf A.F., Perez-Palacios P., Dary M., Brhada F., Pajuelo E. (2015). Prospecting metal-tolerant rhizobia for phytoremediation of mining soils from Morocco using *Anthyllis vulneraria* L.. Environ. Sci. Pollut. Res..

[51] Maynaud G., Willems A., Soussou S., Vidal C., Mauré L., Moulin L., Cleyet-Marel J.-C., Brunel B. (2012). Molecular and phenotypic characterization of strains nodulating *Anthyllis vulneraria* in mine tailings, and proposal of *Aminobacter anthyllidis* sp. nov., the first definition of Aminobacter as legume-nodulating bacteria. Syst. Appl. Microbiol..

[52] Gilbert L.B., Heeb P., Gris C., Timmers T., Batut J., Masson-boivin C. (2010). Experimental evolution of a plant pathogen into a legume symbiont. PLoS Biol..

[53] Vamerali T., Bandiera M., Mosca G. (2010). Field crops for phytoremediation of metal-contaminated land. A review. Environ. Chem. Lett..

[54] Delgadillo J., Lafuente A., Doukkali B., Redondo-Gómez S., Mateos-Naranjo E., Caviedes M.A., Pajuelo E., Rodríguez-Llorente I.D. (2015). Improving legume nodulation and Cu rhizostabilization using a genetically modified rhizobia. Environ. Technol..

[55] Kong Z., Glick B.R., Duan J., Ding S., Tian J., McConkey B.J., Wei G. (2015). Effects of 1-aminocyclopropane-1-carboxylate (ACC) deaminase-overproducing *Sinorhizobium meliloti* on plant growth and copper tolerance of *Medicago lupulina*. Plant Soil.

[56] Ghnaya T., Mnassri M., Ghabriche R., Wali M., Poschenrieder C., Lutts S., Abdelly C. (2015). Nodulation by *Sinorhizobium meliloti* originated from a mining soil alleviates Cd toxicity and increases Cd-phytoextraction in *Medicago sativa* L.. Front. Plant Sci..

[57] Zribi K., Nouairi I., Slama I., Talbi-Zribi O., Mhadhbi H. (2015). *Medicago sativa*—*Sinorhizobium meliloti* Symbiosis Promotes the Bioaccumulation of Zinc in Nodulated Roots. Int. J. Phytoremediat..

[58] Saadani O., Fatnassi I.C., Chiboub M., Abdelkrim S., Barhoumi F., Jebara M., Jebara S.H. (2016). In situ phytostabilisation capacity of three legumes and their associated Plant Growth Promoting Bacteria (PGPBs) in mine tailings of northern Tunisia. Ecotoxicol. Environ. Saf..

[59] Bianucci E., Godoy A., Furlan A., Peralta J.M., Hernández L.E., Carpena-Ruiz R.O., Castro S. (2018). Arsenic toxicity in soybean alleviated by a symbiotic species of *Bradyrhizobium*. Symbiosis.

[60] Chen J., Liu Y.Q., Yan X.W., Wei G.H., Zhang J.H., Fang L.C. (2018). *Rhizobium* inoculation enhances copper tolerance by affecting copper uptake and regulating the ascorbate-glutathione cycle and phytochelatin biosynthesis-related gene expression in *Medicago sativa* seedlings. Ecotoxicol. Environ. Saf..

[61] Zribi K., Djébali N., Mrabet M., Khayat N., Smaoui A., Mlayah A., Aouani M.E. (2012). Physiological responses to cadmium, copper, lead, and zinc of *Sinorhizobium* sp. strains nodulating *Medicago sativa* grown in Tunisian mining soils. Ann. Microbiol..

[62] Fan M., Xiao X., Guo Y., Zhang J., Wang E., Chen W., Lin Y., Wei G. (2018). Enhanced phytoremdiation of *Robinia pseudoacacia* in heavy metal-contaminated soils with rhizobia and the associated bacterial community structure and function. Chemosphere.

[63] Van Houdt R., Mergeay M. (2015). Genomic context of metal response genes in *Cupriavidus metallidurans* with a focus on strain CH34. Metal Response in Cupriavidus Metallidurans.

[64] Rozycki T. Von, Nies Æ.D.H., Alcaligenes W.Á., Ch Á.Á.H. (2008). *Cupriavidus metallidurans*: Evolution of a metal-resistant bacterium. Anton. Leeuwenhoek.

[65] Rubio-Sanz L., Prieto R.I., Imperial J., Palacios J.M., Brito B. (2013). Functional and expression analysis of the metal-inducible *dmeRF* system from *Rhizobium leguminosarum* bv. viciae. Appl. Environ. Microbiol..

[66] Haney C.J., Grass G., Franke S., Rensing C. (2005). New developments in the understanding of the cation diffusion facilitator family. J. Ind. Microbiol. Biotechnol..

[67] Zielazinski E.L., González-Guerrero M., Subramanian P., Stemmler T.L., Argüello J.M., Rosenzweig A.C. (2013). *Sinorhizobium meliloti* Nia is a P1B-5-ATPase expressed in the nodule during plant symbiosis and is involved in Ni and Fe transport. Metallomics.

[68] Li Z., Lu M., Wei G. (2013). An omp gene enhances cell tolerance of Cu(II) in Sinorhizobium meliloti CCNWSX0020. World J. Microbiol. Biotechnol..

[69] Lu M., Li Z., Liang J., Wei Y., Rensing C., Wei G. (2016). Zinc resistance mechanisms of P 1B-type ATPases in *Sinorhizobium meliloti* CCNWSX0020. Sci. Rep..

[70] Lu M., Jiao S., Gao E., Song X., Li Z., Hao X., Rensing C., Wei G. (2017). Transcriptome response to heavy metals in *Sinorhizobium meliloti* CCNWSX0020 reveals new metal resistance determinants that also promote bioremediation by *Medicago lupulina* in metal contaminated soil. Appl. Environ. Microbiol..

[71] Grass G., Rensing C. (2001). CueO is a multi-copper oxidase that confers copper tolerance in *Escherichia coli*. Biochem. Biophys. Res. Commun..

[72] Franke S., Grass G., Rensing C., Nies D.H. (2003). Molecular analysis of the copper-transporting efflux system CusCFBA of *Escherichia coli*. J. Bacteriol..

[73] Long F., Su C.-C., Lei H.-T., Bolla J.R., Do S.V., Yu E.W. (2012). Structure and mechanism of the tripartite CusCBA heavy-metal efflux complex. Philos. Trans. R. Soc. B Biol. Sci..

[74] Kahn D., David M., Domergue O., Daveran M.L., Ghai J., Hirsch P.R., Batut J. (1989). *Rhizobium meliloti* fixGHI sequence predicts involvement of a specific cation pump in symbiotic nitrogen fixation. J. Bacteriol..

[75] Batut J., Terzaghi B., Gherardi M., Huguet M., Terzaghi E., Garnerone A.M., Boistard P., Huguet T. (1985). Localization of a symbiotic *fix* region on *Rhizobium meliloti* pSym megaplasmid more than 200 kilobases from the *nod-nif* region. Mol. Gen. Genet. MGG.

[76] Romaniuk K., Dziewit L., Decewicz P., Mielnicki S., Radlinska M., Drewniak L. (2017). Molecular characterization of the pSinB plasmid of the arsenite oxidizing, metallotolerant *Sinorhizobium* sp. M14—Insight into the heavy metal resistome of sinorhizobial extrachromosomal replicons. FEMS Microbiol. Ecol..

[77] Brokx S.J., Rothery R.A., Zhang G., Ng D.P., Weiner J.H. (2005). Characterization of an *Escherichia coli* sulfite oxidase homologue reveals the role of a conserved active site cysteine in assembly and function. Biochemistry.

[78] Wilson J.J., Kappler U. (2009). Sulfite oxidation in *Sinorhizobium meliloti*. Biochim. Biophys. Acta (BBA) Bioenerg..

[79] Porter S.S., Chang P.L., Conow C.A., Dunham J.P., Friesen M.L. (2017). Association mapping reveals novel serpentine adaptation gene clusters in a population of symbiotic *Mesorhizobium*. ISME J..

[80] Li Z., Ma Z., Hao X., Rensing C., Wei G. (2014). Genes conferring copper resistance in *Sinorhizobium meliloti* CCNWSX0020 also promote the growth of *Medicago lupulina* in copper-contaminated soil. Appl. Environ. Microbiol..

[81] Hao X., Xie P., Zhu Y.-G., Taghavi S., Wei G., Rensing C. (2015). Copper tolerance mechanisms of *Mesorhizobium amorphae* and its role in aiding phytostabilization by *Robinia pseudoacacia* in copper contaminated soil. Environ. Sci. Technol..

[82] Maynaud G., Brunel B., Yashiro E., Mergeay M., Cleyet-Marel J.C., Le Quéré A. (2014). CadA of *Mesorhizobium metallidurans* isolated from a zinc-rich mining soil is a PIB-2-type ATPase involved in cadmium and zinc resistance. Res. Microbiol..

[83] Rubio-Sanz L., Brito B., Palacios J. (2018). Analysis of metal tolerance in *Rhizobium leguminosarum* strains isolated from an ultramafic soil. FEMS Microbiol. Lett..

[84] Pérez-Palacios P., Romero-Aguilar A., Delgadillo J., Doukkali B., Caviedes M.A., Rodríguez-Llorente I.D., Pajuelo E. (2017). Double genetically modified symbiotic system for improved Cu phytostabilization in legume roots. Environ. Sci. Pollut. Res..

[85] Zhang J., Xu Y., Cao T., Chen J., Rosen B.P., Zhao F.-J. (2017). Arsenic methylation by a genetically engineered Rhizobium-legume symbiont. Plant Soil.

[86] Kong Z., Mohamad O.A., Deng Z., Liu X., Glick B.R., Wei G. (2015). Rhizobial symbiosis effect on the growth, metal uptake, and antioxidant responses of *Medicago lupulina* under copper stress. Environ. Sci. Pollut. Res..

[87] Rajkumar M., Ae N., Prasad M.N.V., Freitas H. (2010). Potential of siderophore-producing bacteria for improving heavy metal phytoextraction. Trends Biotechnol..

[88] Valls M., De Lorenzo V. (2002). Exploiting the genetic and biochemical capacities of bacteria for the remediation of heavy metal pollution. FEMS Microbiol. Rev..

[89] Nies D.H. (2003). Efflux-mediated heavy metal resistance in prokaryotes. FEMS Microbiol. Rev..

[90] Drewniak L., Dziewit L., Ciezkowska M., Gawor J., Gromadka R., Sklodowska A. (2013). Structural and functional genomics of plasmid pSinA of *Sinorhizobium* sp. M14 encoding genes for the arsenite oxidation and arsenic resistance. J. Biotechnol..

[91] Drewniak L., Matlakowska R., Sklodowska A. (2008). Arsenite and arsenate metabolism of *Sinorhizobium* sp. M14 living in the extreme environment of the Zloty Stok gold mine. Geomicrobiol. J..

[92] DiCenzo G.C., Finan T.M. (2017). The divided bacterial genome: Structure, function, and evolution. Microbiol. Mol. Biol. Rev..

[93] DiCenzo G.C., Benedict A.B., Fondi M., Walker G.C., Finan T.M., Mengoni A., Griffitts J.S. (2018). Robustness encoded across essential and accessory replicons of the ecologically versatile bacterium *Sinorhizobium meliloti*. PLoS Genet..

[94] DiCenzo G.C., Wellappili D., Golding G.B., Finan T.M. (2018). Inter-replicon gene flow contributes to transcriptional integration in the *Sinorhizobium meliloti* multipartite genome. G3 Genes Genomes Genet..

[95] Landeta C., Dávalos A., Cevallos M.Á., Geiger O., Brom S., Romero D. (2011). Plasmids with a chromosome-like role in Rhizobia. J. Bacteriol..

[96] DiCenzo G.C., Checcucci A., Bazzicalupo M., Mengoni A., Viti C., Dziewit L., Finan T.M., Galardini M., Fondi M. (2016). Metabolic modelling reveals the specialization of secondary replicons for niche adaptation in *Sinorhizobium meliloti*. Nat. Commun..

[97] Galardini M., Brilli M., Spini G., Rossi M., Roncaglia B., Bani A., Chiancianesi M., Moretto M., Engelen K., Bacci G. (2015). Evolution of intra-specific regulatory networks in a multipartite bacterial genome. PLoS Comput. Biol..

[98] Galardini M., Pini F., Bazzicalupo M., Biondi E.G., Mengoni A. (2013). Replicon-dependent bacterial genome evolution: The case of *Sinorhizobium meliloti*. Mol. Biol..

[99] Checcucci A., diCenzo G.C., Ghini V., Bazzicalupo M., Beker A., Decorosi F., Dohlemann J., Fagorzi C., Finan T.M., Fondi M. (2018). Creation and multi-omics characterization of a genomically hybrid strain in the nitrogen-fixing symbiotic bacterium *Sinorhizobium meliloti*. bioRxiv.

[100] Checcucci A., DiCenzo G.C., Bazzicalupo M., Mengoni A. (2017). Trade, diplomacy, and warfare: The quest for elite rhizobia inoculant strains. Front. Microbiol..

[101] Van Opijnen T., Camilli A. (2013). Transposon insertion sequencing: A new tool for systems-level analysis of microorganisms. Nat. Rev. Microbiol..

